# If air conditioning is not functioning…

**DOI:** 10.4103/0019-5049.72657

**Published:** 2010

**Authors:** Medha Mohta

**Affiliations:** Department of Anaesthesiology and Critical Care, University College of Medical Sciences and Guru Teg Bahadur Hospital, Delhi-110 095, India

Sir,

There is a misconception among many of our surgical colleagues that air conditioning in operating theatres is only for our comfort. If it was so, all working areas in the hospitals would have got this facility. But all of us would agree that this is not the case in most of the hospitals in India. This means there has to be much more than just comfort for which air conditioning is required in operating theatres.

Air conditioning is not synonymous with cooling. An optimal air conditioning system filters air, maintains a certain number of air exchanges per hour, and maintains temperature and humidity of the area. Thus, besides providing acceptable indoor climate for the patients and the personnel, air conditioning removes odour and anaesthetic gases, and reduces the risk of infection to the patient by controlling airborne microorganisms in the room. There are standards for operating room air conditioning. Air supply of 0.24 m^3^ per minute per person is the critical level for odour suppression.[1] At least 20 air changes per hour should be maintained to control microorganisms and for comfort. The operation theatre (OT) temperatures should be between 20 and 24°C and the relative humidity 40–60%.[2]

Despite the above facts, which are well mentioned in the books, journals and articles on internet, many surgeons and anaesthetists still get into hot arguments regarding taking up cases in the elective OTs when air conditioning of the hospital is not working. Surgeons are willing to operate even at OT temperatures of 32–34°C. Sometimes they sweat due to excessive heat and the sweat falls into the patient’s wound. Many times, patients become hyperthermic during the surgery. However, the surgeons have a great satisfaction of performing the surgery under adverse circumstances.

Proper air conditioning increases the productivity of staff. Moreover, prevention of hospital acquired infection reduces the cost of antibiotics, hospital stay and loss of productive man hours.[1]. It is very sad that when publications from all over the world comparing different types of air conditioning systems to be used to reduce infection rates are pouring in,[3,4] we, Indians, are still trying to convince each other that air conditioning is mandatory for operating theatres and we should not give in to pressure from our surgical colleagues to allow elective cases to be done if there is a problem with the hospital air conditioning system.
